# Supplementary material for: Understanding the pathogenesis of uveitis in Ebola virus disease survivors: an observational cohort and cross-sectional study protocol for clinical, molecular virologic and immunologic characterisation
Source: BMJ Open. 2025 Dec 23;15(12):e104843. doi: 10.1136/bmjopen-2025-104843 (PMC12730762; doi:10.1136/bmjopen-2025-104843)
Supplement: online supplemental file 3 [file bmjopen-15-12-s003.pdf]

Supplementary File
Ebola Virus Persistence in Ocular Tissues and Fluids Study - Vitreoretinal Surgery (EVICT-VR) Investigators
Study Group Roster

University of Nebraska Medical Center, *Omaha, NE, USA*
Steven Yeh, MD, Christopher D. Conrady, MD, PhD, Caleb Hartley, MPH, Tolulope Fashina, MD, MPH, Angela Hewlett, MD, MS, David Brett-Major, MD, MPH, Michael Wiley, PhD, Ye Huang, MD, Charlene Choo, MD, Shiama Balendra, MBBS, Brent R. Hayek, MD

Ministry of Health National Eye Programme, *Freetown, Sierra Leone*
Lloyd C. Harrison-Williams, MD, John G. Mattia, MD, Jalikatu Mustapha, MD, Matthew J. Vandy, MD

Ministry of Health, *Freetown, Sierra Leone*
Zikan Koroma, MD, Alie Wurie, MD, Theophilus Frankly, Osman Conteh, Benjay, Alicious Kamara, Santigie

Lowell and Ruth Gess Eye Hospital, *Freetown, Sierra Leone*
Ibrahim Conteh, Agnes Konneh, Ken Campbell, Mabel Nanah Sesay, Miatta Dean Rogers, Rosamond Hannah Dowrie, Ramatu Lahai, Mohamed Mansaray, Philip Koroma, Mohamed Kamara, Ibrahim Cotor Bah, Tamba George Mani, Benjamin Williams, Dr. Lloyd Harrison-Wiliams

Kenema Government Hospital Lassa Hemorrhagic Fever Laboratory, *Kenema, Sierra Leone*
Augustine Goba, John D. Sandi, Mambu Momoh, Simbirie Jalloh, Donald S. Grant, MD, MPH

Clinical Monitoring Research Program Directorate, Frederick National Laboratory for Cancer Research, *Frederick, MD, USA*
Ian Crozier, MD

Biosurv International, *Salisbury, England, UK*
Catherine Pratt

Emory University, *Atlanta, GA, USA*
Jessica Carag, DVM, Jill Morgan, RN, BSN, Colleen S. Kraft, MD, MSc, Susanne Linderman, PhD, Carl Davis, PhD, Rafi Ahmed, PhD, Laura Ward, MPH, Sanjana Kuthyar, MD

University of California San Francisco, *San Francisco, CA, USA*
Nisha Acharya, MD, MS, Thuy Doan, MD, PhD, Jessica G. Shantha, MD, MSc

Flinders University, *Adelaide, Australia*
Justine Smith, PhD

Tulane University School of Medicine, *New Orleans, LA, USA*
John S. Schieffelin, MD, Robert F. Garry, PhD

George Mason University, *Fairfax, VA, USA*
Carolyn Drews-Botsch, PhD, MPH

United States Centers for Disease Control and Prevention, *Atlanta, GA, USA*
Timothy M. Uyeki, MD

University of North Carolina-Chapel Hill, *Chapel Hill, NC, USA*
Jean-Claude Mwanza, MD, PhD, MPH

Sierra Leone Association of Ebola Survivors, *Freetown, Sierra Leone*
Mohamed Mansaray, Yusuf Kabba, Daddy Kamara

United States Army Medical Research Institute of Infectious Diseases, *Ft. Detrick, MD, USA*
Xiankun (Kevin) Zeng, PhD

Walter Reed National Army Medical Center, *Washington, D.C., USA*
Grant A. Justin, MD

World Health Organization, *Geneva, Switzerland,*
Anaïs Legand, MPH, Pierre Formenty, DVM, MPH

Central Global Vision Fund, *Millbank, SD, USA*
Roger Reiners, Melanie Reiners
