# Supplementary material for: Understanding the pathogenesis of uveitis in Ebola virus disease survivors: an observational cohort and cross-sectional study protocol for clinical, molecular virologic and immunologic characterisation
Source: BMJ Open. 2025 Dec 23;15(12):e104843. doi: 10.1136/bmjopen-2025-104843 (PMC12730762; doi:10.1136/bmjopen-2025-104843)
Supplement: Supplementary file 4 [file bmjopen-2025-104843.reviewer_comments.pdf]

PEER REVIEW HISTORY

BMJ Open publishes all reviews undertaken for accepted manuscripts. Reviewers are asked to complete a checklist review form and are provided with free text boxes to elaborate on their assessment. These free text comments are reproduced below.

ARTICLE DETAILS

Title (Provisional)

Understanding the pathogenesis of uveitis in Ebola virus disease survivors: an observational cohort and cross-sectional study protocol for clinical, molecular virologic, and immunologic characterization.

Authors

Hartley, Caleb D.; Linderman, Susanne; Fashina, Tolulope; Ward, Laura; Drews-Botsch, Carolyn; Pratt, Catherine; Kuthyar, Sanjana; Fernandes, Alcides Filho; Huang, Ye; Choo, Charlene; Nguyen, Nam; Carag, Jessica; Morgan, Jill; Kraft, Colleen S; Hewlett, Angela; Brett-Major, David; Schieffelin, John S.; Garry, Robert F; Grant, Donald; Justin, Grant A.; Conrady, Christopher D.; Smith, Justine R.; Hayek, Brent R.; Balendra, Shiama; Acharya, Nisha; Doan, Thuy; Legand, Anais; Formenty, Pierre; Zeng, Xiankun; Conteh, Ibrahim; Vandy, Matthew Jusu; Harrison-Williams, Lloyd; Mustapha, Jalikatu; Koroma, Zikan; Wiley, Michael R; Crozier, Ian; Mwanza, Jean-Claude; Shantha, Jessica G; Ahmed, Rafi; Yeh, Steven; SMILE and EVICT-VR, Investigator Study Groups

---

VERSION 1 - REVIEW

**Reviewer**	**1**
**Name**	**Burkholder, Bryn M.**
**Affiliation**	**Johns Hopkins University**
**Date**	**04-Jul-2025**
**COI**	**None**

The authors should be commended for this well-designed study and well-written report.

**Reviewer**	**2**
**Name**	**Usui, Yoshihiko**
**Affiliation**	**Tokyou Medical University Hospital, Ophthalmology**
**Date**	**07-Sep-2025**
**COI**	**None**

This protocol presents a well-motivated, multi-aim study to characterize EVD-associated intraocular inflammation (EVD-IOI) through epidemiology (SMILE cohort), ocular virology/immunology in surgical specimens (EVICT-VR), and systemic immune profiling of survivors with/without uveitis. The abstract clearly states the clinical importance, the uncertainty around viral persistence and host responses, and the plan to address these knowledge gaps. The three aims and their relationships (SMILE, EVICT-VR, and immune phenotyping) are laid out with appropriate collaborations across ophthalmology, infectious disease, and immunology.

1) The Aim 1 case definition of EVD-associated uveitis is broad ("evidence of current or prior uveitis and/or its sequelae…during or after EBOV infection, not related to other causes"). While pragmatic, this could misclassify incident uveitis unrelated to prior EVD—particularly in a region with other infectious uveitides. Please consider: (i) adjudication by a masked uveitis panel; (ii) explicit operationalization of SUN criteria for anterior/intermediate/posterior/panuveitis; and (iii) a standardized rule-out panel (e.g., syphilis serology, TB screening as feasible) to minimize non-EVD uveitis misclassification. The current text defines the case concept and lists included signs, which is helpful, but more operational detail would strengthen internal validity.

2) Household "close contacts" may be anti-EBOV IgG–positive and are analyzed separately, which is sensible; however, there's a typographical artifact "anti-EBOV-X IgG" that should be corrected. Also, for clarity, specify a priori how seropositive contacts will be handled in the primary vs. sensitivity analyses and whether matching/adjustment for age/sex/exposure will be used to mitigate confounding.

3) The calculation assumes 10% uveitis in survivors vs 1% in controls, yielding n=200/group for >80% power at 95% confidence. Please provide citations or prior data supporting the 10% vs 1% difference, and clarify handling of clustering (household contacts) if any. Also note that the protocol plans a 1:2 survivor:contact ratio (≥250 survivors, 500 contacts); please reconcile analysis plans (unadjusted vs adjusted) with this ratio and any site or household-level effects.

4) Aim 2 will collect 1.0–1.5 mL undiluted vitreous at PPV to test EBOV RNA and calculate Goldmann-Witmer Coefficient (GWC). Please pre-specify: (i) surgical-indication matching between EVD survivors and non-EVD uveitis controls to avoid spectrum/selection bias; (ii) analytical thresholds (e.g., GWC >3 as positive) and how breakdown of the blood-ocular barrier will be accounted for; and (iii) the LoD, Ct thresholds, and inhibition controls for RT-qPCR (particularly on viscous vitreous) as this will directly influence the expected proportion of RNA positives.

5) The plans for bivariate tests and multivariable models across aims are appropriate; however, multiple testing is likely (e.g., numerous immune markers in Aim 3). Please specify the approach to multiplicity control (e.g., FDR) and define model covariates a priori, including how "time from acute EVD" will enter models (continuous vs categories/splines).

Also, “Jeffreys interval” is misspelled as “Jeffrey’s” in the text—please correct and provide a brief rationale for this interval choice.

6) The imaging suite (color photography, OCT, FFA, B-scan) is comprehensive. Please add: (i) standardized acquisition protocols; (ii) a masked central reading process; and (iii) inter-grader reliability plans (e.g., kappa/ICC), which will be essential for reproducible phenotyping and severity metrics.

7) The antigen-specific tetramers and intracellular cytokine staining are cutting-edge, as is repertoire sequencing from ocular samples. Please clarify: (i) antigen targets (e.g., GP, NP) and peptide pools; (ii) sample processing/cryopreservation and shipping conditions from Sierra Leone; and (iii) how T/B-cell clonotypes detected in vitreous will be declared “EBOV-specific” (matching criteria to reference clonotypes) versus bystander/expanded clones.

8) The high-level IPC measures modeled on prior EVICT work are appropriate; the protocol would benefit from naming the safety monitoring entity (e.g., internal safety board) and the specific thresholds for pausing procedures (e.g., complication rates) beyond the qualitative “increase in complications.” Add an escalation workflow and documentation cadence.

9) The DFnet/DFcollect pipeline, validation, and edit-check processes are well described. Please add: (i) a data dictionary and variable provenance plan; (ii) role-based access with audit trails; (iii) long-term retention, and (iv) a clear data-sharing statement consistent with BMJ Open policy (e.g., de-identified datasets, timelines, and request mechanism), as only security/hosting is currently specified.

10) Patients/public were not involved. Given the central role of SLAES, consider a modest PPI component (e.g., co-developed lay summaries, feedback on visit burden) and a dissemination plan targeted to survivor communities and the MOH.

11) Recruitment was planned to complete by 31 July 2025; now is September 2025 (JST). Please update status (accrual numbers by arm, reasons for delays if any) and revise downstream milestones (e.g., completion of immune profiling by March 2026).

12) IRB approvals are clearly listed for UNMC, Emory, and Sierra Leone MOH. Add any material amendments since initial approval (e.g., expanded inclusion, assay updates) and ensure the consent forms explicitly address incidental findings and post-operative care.

13) “anti-EBOV-X IgG” → “anti-EBOV IgG.”

14) “EVD survivior” → “EVD survivor.” (Aim 2 inclusion text)

15) “Jeffrey’s interval” → “Jeffreys interval.”

VERSION 1 - AUTHOR RESPONSE

Reviewer 1 Comment

Reviewer's Comment: The authors should be commended for this well-designed study and well-written report.

Author's Response: Thank you for this comment and time in reviewing our manuscript. We greatly appreciate the positive feedback on the study design and the clarity of our report.

Reviewer 2 Comments

1. The Aim 1 case definition of EVD-associated uveitis is broad ("evidence of current or prior uveitis and/or its sequelae…during or after EBOV infection, not related to other causes"). While pragmatic, this could misclassify incident uveitis unrelated to prior EVD—particularly in a region with other infectious uveitides. Please consider: (i) adjudication by a masked uveitis panel; (ii) explicit operationalization of SUN criteria for anterior/intermediate/posterior/panuveitis; and (iii) a standardized rule-out panel (e.g., syphilis serology, TB screening as feasible) to minimize non-EVD uveitis misclassification. The current text defines the case concept and lists included signs, which is helpful, but more operational detail would strengthen internal validity.

Author's Response: Thank you for this thoughtful comment. We agree that precise case definition is critical for internal validity. Our study protocol specifies that uveitis will be classified using the Standardization of Uveitis Nomenclature (SUN) criteria, with categorization into anterior, intermediate, posterior, and panuveitis. In addition, as described in the Methods section, participants with evidence of uveitis undergo laboratory testing for syphilis, tuberculosis, HIV, and toxoplasmosis when clinically indicated, to reduce misclassification from other infectious causes. We agree that masked adjudication by a uveitis panel could further strengthen case definition. While this is not part of the current study design, we recognize its value and will incorporate adjudication approaches in future research as our study infrastructure and personnel develop further.

2. Household "close contacts" may be anti-EBOV IgG–positive and are analyzed separately, which is sensible; however, there's a typographical artifact "anti-EBOV-X IgG" that should be corrected. Also, for clarity, specify a priori how seropositive contacts will be handled in the primary vs. sensitivity analyses and whether matching/adjustment for age/sex/exposure will be used to mitigate confounding.

Author's Response: Thank you for noting this error. Line 240 in the revised manuscript, has been revised to reflect the correct term "anti-EBOV IgG. Corrected "anti-EBOV-X IgG" → "anti-EBOV IgG." We also clarified in the manuscript that seropositive contacts will be analyzed as a separate group in the primary analysis, with sensitivity analysis excluding

them. We added that multivariable analysis will adjust for ae, sex, and exposure.

3. The calculation assumes 10% uveitis in survivors vs 1% in controls, yielding n=200/group for >80% power at 95% confidence. Please provide citations or prior data supporting the 10% vs 1% difference and clarify handling of clustering (household contacts) if any. Also note that the protocol plans a 1:2 survivor: contact ratio (≥250 survivors, 500 contacts); please reconcile analysis plans (unadjusted vs adjusted) with this ratio and any site or household-level effects.

Author's Response: We have added citations (Mattia et al. 2016; PREVAIL III, 2019; Shantha et al. 2017) to support the estimated 10% vs 1% uveitis prevalence assumption in the "Patient Recruitment Strategy for Research Aims" section (Lines 218-219) of the revised manuscript. We will incorporate multivariate logistic regression models for the primary outcome (i.e., uveitis versus no uveitis) and multivariate linear regression models for key secondary outcomes including logMAR visual acuity to assess for risk factors for uveitis and better/ poorer visual acuity outcomes, respectively. Unadjusted and adjusted estimates will be represented in tables with robust methods for model development. This will incorporate the Reviewers' important consideration re: the nuance of household level effects, which we have also considered. Two options we have considered are the employment of multilevel / hierarchical models with household as a random effect vs. use of a general estimating equation as a clustering unit. Within the body of the text, we have discussed the household adjustment given the potential for shared risk factors (i.e., housing conditions, shared diet, other infectious exposures).

4. Aim 2 will collect 1.0–1.5 mL undiluted vitreous at PPV to test EBOV RNA and calculate Goldmann-Witmer Coefficient (GWC). Please pre-specify: (i) surgical-indication matching between EVD survivors and non-EVD uveitis controls to avoid spectrum/selection bias; (ii) analytical thresholds (e.g., GWC >3 as positive) and how breakdown of the blood-ocular barrier will be accounted for; and (iii) the LoD, Ct thresholds, and inhibition controls for RT-qPCR (particularly on viscous vitreous) as this will directly influence the expected proportion of RNA positives.

Author's Response: Thank you for this thoughtful comment about a number of variables that impact (i) selection of surgical patients, (ii) analytic thresholds and (iii) Ct LoD. (i) For the surgical indication matching, ideally, we would be able to match the indications in EVD survivors (e.g., intermediate uveitis patient with vitreous opacity and epiretinal membrane) with a non-EVD uveitis patient with the same indication. Because patients who require vitreoretinal surgery who fit the categories will be offered surgery and protocol enrollment, we will account for these varying indications in the statistical analyses where possible. However, the challenge will be sample sizes, logistics, and the level of funding required for a larger sample size that would help to reduce bias. More specifically, if we were to match indications across groups, the number of patients needed to screen would exceed what is budgetarily feasible at this point, although we agree this would be a more rigorous approach.

To limit selection bias for the future, we would assess our initial cohort of ~25-30 patients and review the number of patients screened and disease indications, as a metric of how many patients we would need to screen for a larger study. In a larger study, we would anticipate selection of consecutive patients with a surgical indication by group (e.g., n=5 retinal detachment patients / group; n=5 epiretinal membranes / group, etc). At present, patients will be recruited from the Ministry of Health National Eye Programme and Sierra Leone Association of Ebola Survivors (SLAES). We will acknowledge the potential for selection bias in the early phase of the surgical study in future publications.

(ii) The literature has varied regarding a positive GWC from 2-3 as the cutoff for a positive result. Because uveitis patients can have protein egress from the serum via breakdown of the blood-ocular barrier, as mentioned by the Reviewer, we plan to compare Ebola antigen-specific antibody to influenza antigen-specific antibody relative to serum, as influenza is rarely associated with ocular disease. Elevation of Ebola antigen-specific antibody compared to influenza antigen-specific antibody would be helpful to understand local antigen targeting irrespective of blood-ocular barrier considerations although this is difficult to fully correct given that there will be patient-to-patient variability in blood-ocular barrier breakdown. If the Reviewers have alternative suggestions, we would welcome further opportunities to account for this anatomic change that is observed in uveitis / ocular inflammatory disease.

(iii) qRT-PCR for Ebola will be performed with NP and GP targets with a CT value < 40 and LoD < 10 by either of two EBOV antigen-targeting assays, with MS2 phage, or alternate, internal control to monitor for inhibition.

5. The plans for bivariate tests and multivariable models across aims are appropriate; however, multiple testing is likely (e.g., numerous immune markers in Aim 3). Please specify the approach to multiplicity control (e.g., FDR) and define model covariates a priori, including how "time from acute EVD" will enter models (continuous vs categories/splines). Also, "Jeffreys interval" is misspelled as "Jeffrey's" in the text—please correct and provide a brief rationale for this interval choice.

Author's Response: Thank you for this comment. Line 402 of the revised manuscript have been revised to spell "Jeffreys interval correctly". The Jeffreys interval approach has been chosen given its advantage of being equal-tailed for a 95% confidence interval (vs. Wilson's interval approach.). We have added language re: false discovery rate controls to minimize the chance of false positives for multiple testing (e.g., Benjamini-Hochberg FDR or Benjamini-Yekutieli for correlated exposures).

6. The imaging suite (color photography, OCT, FFA, B-scan) is comprehensive. Please add: (i) standardized acquisition protocols; (ii) a masked central reading process; and (iii) inter-grader reliability plans (e.g., kappa/ICC), which will be essential for reproducible phenotyping and severity metrics.

Author’s Response: Thank you for this suggestion. We have added description of standardized acquisition protocols, centralized masked reading center, and inter-grader reliability testing (kappa/ICC) to the study procedures section of the manuscript under Aim 1 (lines 326-330)

7. The antigen-specific tetramers and intracellular cytokine staining are cutting-edge, as is repertoire sequencing from ocular samples. Please clarify: (i) antigen targets (e.g., GP, NP) and peptide pools; (ii) sample processing/cryopreservation and shipping conditions from Sierra Leone; and (iii) how T/B-cell clonotypes detected in vitreous will be declared “EBOV-specific” (matching criteria to reference clonotypes) versus bystander/expanded clones.

Author’s Response:
Thank you for this comment and we agree with the importance of antigen-specific tetramer and intracellular cytokine staining. For the (i) antigen targets of antigen-specific tetramers and peptide pools we will target Ebola GP, NP, and VP40.

(ii) Sample processing for EBOV antigen-specific ELISA testing will be performed in Sierra Leone. The specimens will be maintained within a temperature monitored -80 degrees C freezer and shipped in dry ice, with replenishment as needed as the specimen is transported. All specimen transportation will adhere to export and important guidance according to country-level and regulatory guidance for biological materials.

(iii) To detect T/B-cell clonotypes in the vitreous which are “EBOV-specific”, we will compare vitreous derived T and B cell receptor sequences to the sequences of Ebola tetramer positive T cells and Ebola GP probe specific B cells derived from the blood. Additionally, we will confirm B cell specificity by recombinant expression of antibodies.

8. The high-level IPC measures modeled on prior EVICT work are appropriate; the protocol would benefit from naming the safety monitoring entity (e.g., internal safety board) and the specific thresholds for pausing procedures (e.g., complication rates) beyond the qualitative “increase in complications.” Add an escalation workflow and documentation cadence.

Author’s Response: Thank you for your comment. We have added to the “Safety Considerations and Monitoring” section, that the safety oversight will be conducted by an internal Data and Safety Monitoring Committee consisting of senior investigators and clinical leads. We have also stated that procedures will be re-evaluated if serious complication rates exceed 10%, with formal escalation workflows.

9. Comment #9: The DFnet/DFcollect pipeline, validation, and edit-check processes are well described. Please add: (i) a data dictionary and variable provenance plan; (ii) role-based access with audit trails; (iii) long-term retention, and (iv) a clear data-sharing statement consistent with BMJ Open policy (e.g., de-identified datasets, timelines, and request mechanism), as only security/hosting is currently specified.

Author’s Response: We have expanded the Data Monitoring section of the manuscript to include data dictionary, variable provenance, role-based access, audit trails, retention policies, and BMJ-compliant data sharing statement.

10. Patients/public were not involved. Given the central role of SLAES, consider a modest PPI component (e.g., co-developed lay summaries, feedback on visit burden) and a dissemination plan targeted to survivor communities and the MOH.

Author’s Response: Thank you for this thoughtful comment. Although patients were not directly involved in the design phase, the Sierra Leone Association of Ebola Survivors (SLAES) will be engaged to provide feedback on study procedures and their associated burden. SLAES representatives will co-develop lay summaries of findings and participate in dissemination activities targeting survivor communities. We have revised the PPI section of the manuscript to reflect this.

11. Recruitment was planned to complete by 31 July 2025; now is September 2025 (JST). Please update status (accrual numbers by arm, reasons for delays if any) and revise downstream milestones (e.g., completion of immune profiling by March 2026).

Author’s Response: Since the manuscript was submitted in May 2025, no additional participant recruitment has occurred. Study activities during this period have focused on operational aspects, including stakeholder engagement, training of study staff, sample storage and transportation logistics, as well as quality assurance procedures. Recruitment is now planned to be completed by December 2025. Downstream milestones, including completion of immune profiling, remain on track as previously described.

12. IRB approvals are clearly listed for UNMC, Emory, and Sierra Leone MOH. Add any material amendments since initial approval (e.g., expanded inclusion, assay updates) and ensure the consent forms explicitly address incidental findings and post-operative care.

Author’s Response: Since initial approval, no amendments to expand inclusion criteria or update immunologic assays have been obtained. Both the UNMC IRB and the Sierra Leone Ministry of Health Ethics Committee conduct annual continuing reviews of the study protocol, during which we provide updates on study timelines and recruitment progress. We thank the reviewer for the suggestion regarding the informed consent forms and will continue to ensure that all participant materials remain current and appropriate.

13. anti-EBOV-X IgG” → “anti-EBOV IgG.”

Author’s Response: Thank you for noting this error. Line 240 in the manuscript have been revised to reflect the correct term “anti-EBOV IgG”

14.“EVD survivior” → “EVD survivor.” (Aim 2 inclusion text)

Author’s Response: Thank you for noting this error. Line 251 in the inclusion text of Aim 2 has been revised to reflect the correct spelling “EVD survivior”.

15. “Jeffrey’s interval” → “Jeffreys interval.”

Author’s Response: Thank you for noting this error. Line 402 of the revised manuscript has been revised to reflect the correct term “Jeffreys interval”.
